# Implementing ecological momentary assessments to measure violence and adolescent HIV transmission risk: Lessons from Johannesburg, South Africa

**DOI:** 10.1371/journal.pdig.0000283

**Published:** 2024-02-02

**Authors:** Janan Janine Dietrich, Stefanie Hornschuh, Phumla Madi, Candice W. Ramsammy, Lerato Tsotetsi, Gugulethu Tshabalala, Busisiwe Nkala-Dlamini, Avy Violari, Rachel Kidman

**Affiliations:** 1 Perinatal HIV Research Unit (PHRU), Faculty of Health Sciences, University of the Witwatersrand, Johannesburg, South Africa; 2 Health Systems Research Unit, South African Medical Research Council, Bellville, South Africa; 3 African Social Sciences Unit of Research and Evaluation (ASSURE), a division of the Wits Health Consortium, University of the Witwatersrand, Johannesburg, South Africa; 4 Department of Social Work, Faculty of Humanities, University of the Witwatersrand, Johannesburg, South Africa; 5 Program in Public Health, State University of New York at Stony Brook, Stony Brook, New York, United States of America; 6 Department of Family, Population and Preventive Medicine, State University of New York at Stony Brook, Stony Brook, New York, United States of America; Technical University Munich, GERMANY

## Abstract

Ecological Momentary Assessment (EMA) is an important methodology to understand risky behaviour and holds promise for HIV research. EMA is still novel in sub-Saharan Africa. We describe challenges and lessons learned on a novel study implementing mobile phone EMAs with adolescent boys in South Africa. The Tsamaisano study was a longitudinal study from 2020–2023 to recruit adolescent boys aged 15–19 years; including those without HIV and those perinatally infected and living with HIV. Participants were prompted to complete 52 weekly mobile phone survey on emotional state, exposure to and perpetration of violence, and sexual risk behaviour. Surveys were delivered using a random algorithm to choose the day. We incorporated mechanisms to assess challenges and optimize survey completion: weekly team meetings with youth representation and real-time data monitoring. Additionally, 20 frequent vs infrequent survey submitters participated in qualitative interviews about barriers and recommendations. Real-time monitoring indicated low (defined as <50%) survey completion in the first months of study implementation. To ensure that both the adolescent participant and their caregiver understood the commitment required for successful EMA, we created and implemented a guided discussion around mobile phone access during the enrolment visit. We identified a need for increased and ongoing technical support; addressed by creating technical guides, implementing a standard two-week check-in call after enrolment, adding an automated request button for call-back assistance, creating a WhatsApp messaging stream, and reaching out to all participants failing to submit two sequential surveys. Entry-level smartphones, including those initially distributed by the study, did not have capacity for certain updates and had to be replaced with more expensive models. Participants struggled with randomly allocated survey days; completion improved with set completion days and targeted reminder messages. Together, these steps improved survey completion from 40% in December 2020 to 65% in April 2022. We describe key lessons learned to inform future study designs with mobile phone EMAs, drawing on our experience implementing such among adolescent boys, including persons living with HIV, in a low-and-middle income setting. The key lessons learned through the Tsamaisano study are important to inform future study designs with EMA utilizing mobile phone, electronic data collection among adolescent boys in low-and-middle-income settings.

## Author summary

In this study, participants completed 52 weekly mobile phone surveys on emotional state, exposure to and perpetration of violence, and sexual risk behaviour. Although our data are restricted to adolescent boys, lessons can be applied to youth in low-income South African settings. Our study started with randomly selected survey submission days and then switched to set weekly survey days. We incorporated youth engagement strategies through working closely with the Adolescent Community Advisory Board (ACAB) of the PHRU throughout the study implementation, including youth representation as of the study operations meetings.

## Introduction

Ecological Momentary Assessment (EMA) is an important tool to capture how risk behavior develops in real-time. EMA is typically characterized by repeat, self-report assessments that take place under natural conditions [1,2]. EMA thus limits recall bias and provides results that are more accurate to the subject’s environment [3]. In its original form, EMA methods involved participants recording their experiences in a paper-based diary format [1,4]. Increasingly, data capturing is moving towards technology-enabled platforms, a process that was accelerated during the COVID-19 pandemic [5]. Most people now have access to a mobile phone [6], which further provides unique opportunities for mobile data collection, such as updating the survey and allowing for monitoring of large volumes of incoming data in real time, [7–9].

EMA has been successfully implemented to study physical activity, mental health (mood disorders and substance abuse), eating behaviours, and chronic pain, most frequently among adult populations [10]. Among young people specifically, EMA studies have been conducted in high income settings to assess physical activity, substance abuse (particularly smoking) and mental health [10,11]. Risk behaviour assessments remain a challenge in HIV research. However, its use within the HIV field is growing. In the US, a recent literature review on EMA in HIV research identified EMA methods using smartphone apps for HIV primary prevention, HIV treatment adherence, and substance use among young adults and adult populations [12]. Notably, previous South African research utilising daily mobile phone EMA measures was conducted among young women 18–24 years to assess HIV risk and showed more accurate reporting via the mobile phone when compared to the in-clinic survey measure [13–16].

EMA designs hold great promise, but researchers are still learning how best to implement them to collect quality data [17,18]. For example, a recent review noted challenges, such as incompatible smartphones and poor network connectivity, that reduced compliance [10,18]. A systematic review evaluating the feasibility of using EMA for youth psychopathology found that the main barrier to success was overburdening participants with submissions. The authors recommended adapting the methods to the specific population group to encourage compliance [11]. For example, the survey questions should be clear and easily understandable to avoid misinterpretation and take the literacy levels of participants into account. Furthermore, suggestions to increase compliance included the use of phones that are compatible to the data collection program used [19].

EMA could yield important insights in the relationships between HIV and violence. Both are endemic in South Africa [20]: intimate partner violence (IPV) and other types of victimization are widely recognized as key drivers of HIV transmission [21–24]. Adolescent boys living with HIV and who are perinatally infected may be more vulnerable to violence. This is because many have lost a parent to AIDS, which increases the likelihood of childhood sexual abuse by approximately 50% [25] and also HIV disclosure can trigger violent reactions from partners or peers [26]. Engagement in violence can trigger risky sexual behaviours among young men including multiple partners, condom use, drugs and alcohol abuse and transactional sex [27].

We designed the Tsamaisano study to better understand how violence shapes risk behaviors among adolescent boys in South Africa, utilizing a mobile EMA approach. As we were one of the first studies to use EMA to measuring sensitive topics among adolescents in this setting, we reviewed the literature for best practices. However, EMA is relatively novel in sub-Saharan African settings, and there was limited guidance on how to develop and implement EMA methods in these unique contexts. Within the main study, we thus embedded processes that would allow for continuous quality improvement. The aim of this paper is to describe the lessons learned with recommendations for smoother EMA data collection. This might benefit other researchers who intend to employ similar strategies with populations, such as adolescent boys, that are usually hard to engage in clinic and health settings [28].

## Methods

### Tsamaisano study

The Tsamaisano study was a prospective longitudinal study (2020–2024) to understand how violence influences HIV transmission risk. Participants included adolescent boys aged 15–19 years; those without HIV and persons living with HIV through perinatal infection. The study took place in Soweto, South Africa, a densely populated and large urban settlement or township of about 1.7 million inhabitants [29]. Eligible participants are: male; 15–19 years at baseline; living in Soweto; and either in a current dating relationship with someone they see face-to-face or have had sex in the last month. Inclusion criteria for persons living with HIV included perinatal HIV infection (defined as having had a history of HIV infection before age 10 years) and awareness of their HIV diagnosis. Participant exclusion criteria included acute psychiatric illness or cognitive impairment rendering them unable to perform the weekly surveys.

Following recruitment (in the community and in HIV clinics), prospective participants were invited to an in-person enrolment visit. The visit was conducted at the Perinatal HIV Research Unit (PHRU) [30], located at the Chris Hani Baragwanath Academic Hospital. At this visit participants completed an interactive survey training session. Participants were told they would get a text request to complete a survey on their mobile phones once a week for 52 weeks. The mobile phone survey, hereafter referred to as the survey, was designed within the SurveyCTO application (app) [31] an online survey platform, and data were managed by ikapadata [32], a local South African digital health company.

### Initial literature review

Prior to study initiation, a brief literature search was conducted to review strategies shown to help retain youth in mobile phone-based research, including but not limited to EMA. Previous research among youth in Soweto showed more than 90% of young people aged 14–19 years owned a mobile phone, and 65% had access to the internet [33,34]. Therefore, we planned to provide entry level android smartphones to 20% of participants enrolled in the study. Previous qualitative research on mobile phone-based data collection conducted among youth in Soweto indicated a preference for short surveys that were easy to answer, with mobile phone data provided to participants to access the applications for survey completion, followed by an airtime incentive, a voucher that could be redeemed immediately for mobile phone airtime, after survey submission [13–15]. Additionally, reminder messages were recommended for repeated EMA mobile phone based surveys [14].

### Patient and Public Involvement: Youth Engagement before the study start

To ensure a youth engaged strategy, the study team worked closely with the Adolescent Community Advisory Board (ACAB) of the PHRU throughout the study implementation. The ACAB is an important bridge back to the community. We hosted a workshop at the beginning of the project to inform them of study details to get feedback on study development. The ACAB is comprised of approximately 16 youth, 16–23 years and from different organisations and schools in and around Soweto. The members of the ACAB were consulted and involved in the formulations of the initial survey questions. This was to ensure that questions were developmentally and culturally appropriate for male adolescents, understandable in English and translated appropriately to the local languages (isiZulu and Sesotho). The ACAB advised on recruitment strategies and continue to advise the study team throughout the study period.

The survey was pilot tested among 10 adolescent boys 15–19 years, including those living with HIV and those living without HIV infection. Potential participants were recruited from Soweto and signed informed consent to participate in weekly surveys over a four week period. Following their participation, all pilot participants were interviewed telephonically for feedback on their experiences and suggestions to improve implementation.

### Survey structure and training with participants

**Fig 1:**
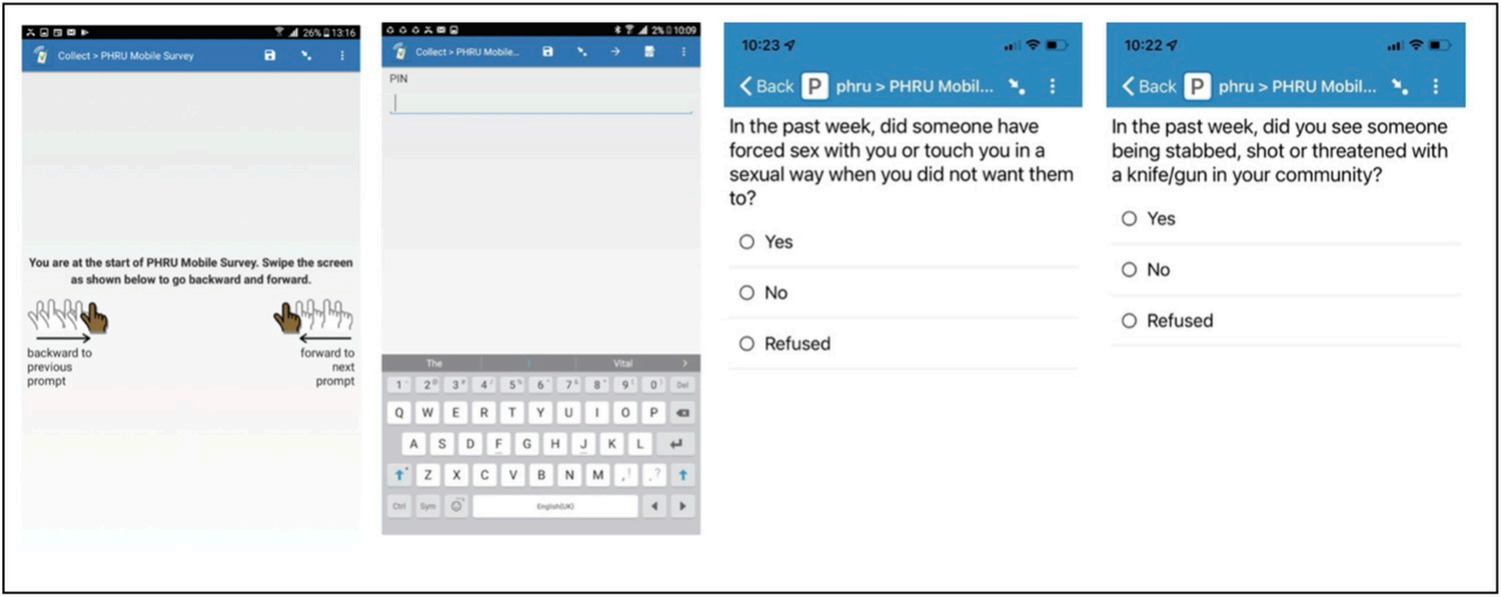
Example mobile phone survey screens and questions.

The survey (See Fig 1) included 23 and 25 questions for those living without HIV infection and for persons living with HIV, respectively. The survey included items on mental health (depression, anxiety), alcohol use, physical and sexual violence exposure, intimate partner violence (IPV) perpetration [35–37], sexual activity, condom use. Additional questions on antiretroviral treatment adherence were included for persons living with HIV. Questions on COVID-19 (for example, symptoms, and testing) were also included once a month.

Entry-level smartphones were provided to participants who did not have their own phones or those whose phones did not meet the requirements to download and run the SurveyCTO app [31]. Participants could keep these study phones upon study completion. During study enrolment, mobile phone educators helped participants install the SurveyCTO app on their phones and demonstrated how to complete the survey. Participants were provided with a printed manual on the SurveyCTO app set up and survey completion. In addition, a once-off automated SMS with their individual PIN to access the survey and a link to the survey manual was sent to the participant’s phone. Both the survey and the manual are available in three languages, English, isiZulu and Sesotho.

Surveys started the Monday following study enrolment. Data to the value of 100MB is preloaded on the phones in the afternoon (15:00) of the day a survey request is made through an online app programming interface SIM control [38]. The preloaded data was to ensure that participants would (1) not incur any costs and (2) have sufficient data to be able to submit their survey. An automated survey request SMS was initiated at 15:00 on the day of the participant’s expected submissions day; a reminder SMS followed at 19:00 that day as well as at 15:00 the following day, if the survey was not submitted. Different survey request and reminder SMS wordings were generated in collaboration with the ACAB and were randomly sent out for each time point. Following successful survey submission, an automated confirmation SMS was sent. Participants received a 10ZAR (~70 US cents) airtime voucher incentive if submitted within a two-day window after the initial request. Participants were contacted by the mobile phone educators telephonically and/or via WhatsApp after completing the first survey to troubleshoot any technical problems.

### Continuous quality improvement strategies

Weekly team meetings were held to review study progress review survey completion rates and to discuss challenges of implementation. At the time, the EMA data collection was a new data collection technique incorporating sensitive questions. Thus, the monitoring of survey completion rates during the team meetings and a rapid qualitative evaluation were critical to establish barriers to survey completion. Following a text message prompt to participants, a successful survey submission was defined uploading the survey in the SurveyCTO app on the same day or within a 2-day period.

The qualitative evaluation included in-depth interviews with 20 of the Tsamaisano participants attending a follow-up visit 3–6 months after enrolment. The sample size was sufficient to reach data saturation [13,15]. A Tsamaisano staff member received a list of pre-selected participants based on the month of enrolment, survey performance, age group and HIV status. Interviewers conducted a 30–60 minute in-depth interview in a private room at the PHRU.

Participants signed a separate informed consent or assent form for participation in the interviews. Participants were able to select a trained male or female multilingual interviewer. A semi-structured interview guide addressed topics on overall study participation experience, barriers and facilitators to survey completion, and recommendations for improving the survey implementation. All interviews were audio-recorded, transcribed to English, and uploaded in Nvivo [19]. Data were coded and analysed by three coders using a framework data analysis approach [20,21]. First transcripts were read for data familiarisation, then the same initial coder developed a codebook a-priori for barriers to survey completion and recommendations for improvement. Participant demographic characteristics included age, work and income, mobile phone ownership and use, and internet use. Descriptive statistics were performed to calculate frequencies and percentages by HIV status using Stata version 17 (Stata Corp).

### Ethical considerations

All study procedures were approved by the Institute Research and Ethics Committees of the University of the Witwatersrand, South Africa (Approval ID: 191001) and Stony Brook University, United States (Approval ID: FWA#00000125 and IRB2019-00567). Written study informed consent and assent for participants younger than 18 years were obtained from all participants. Parents/legal guardians provided written informed consent for participants younger than 18 years. Participants and parent/legal guardian signed separate forms for audio-recording for in-depth interviews. All methods were carried out in accordance with relevant guidelines and regulations. All changes made to improve survey submission rates were approved by Ethics Committees of the University of the Witwatersrand and Stony Brook University. Participants were reimbursed R250 (~ $16) for scheduled visits, which for some included qualitative interviews. A PHRU counsellor was available for those requiring psychosocial support.

## Results

### Survey completion

**Fig 2:**
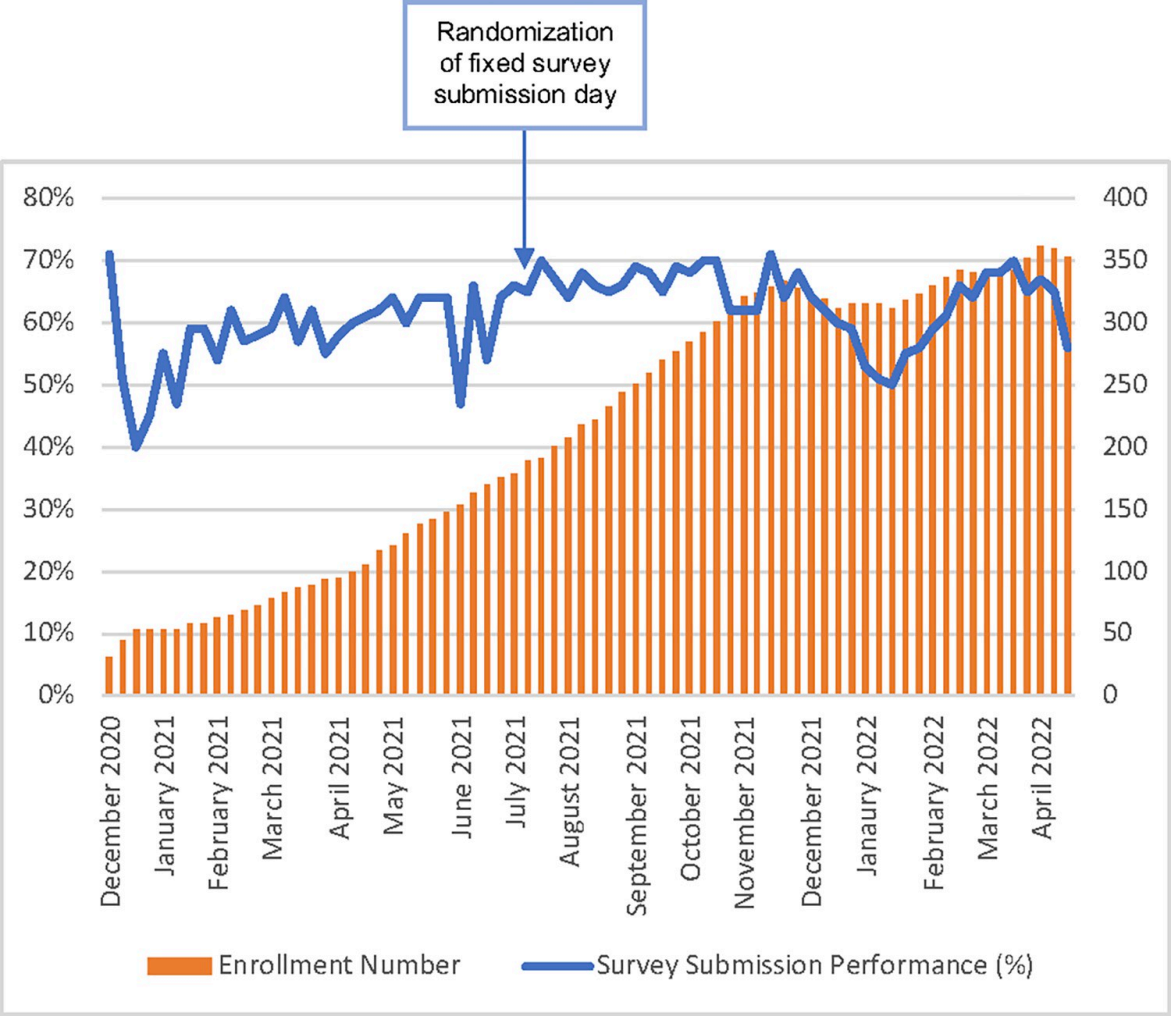
Survey submission performance between December 2020 and April 2022.

Enrolment started in November 2020, prior to the second COVID-19 wave in South Africa. From November 2020—April 2022, 468 participants were enrolled; 241 persons living with HIV (median age was 16 (15–19)), 227 without HIV (16 (15–19)) (Table 1). Fig 2 shows overall survey performance. We define successful completion as submitting a survey via the SurveyCTO app with 48 hours of the request or after of a given week and transmission thereof into the study database. Since study start, the study team closely monitored participants’ survey submission rates and identified that the submission rates were below 50%. Our continuous quality improvement process helped increase survey response rates from 40% in December 2020 to 65% by April 2022.

**Table 1:** Demographic characteristics.

Variable	All n = 468 n(%)	Persons living with HIV n = 241 n(%)	Persons without HIV n = 227 n(%)	p-value
**Age**				
15–17	352 (75.9)	177 (74.4)	175 (77.4)	0.441
18–19	112 (24.1)	61 (25.6)	51 (22.6)	
Median (range)	16 (15–19)	16 (15–19)	16 (15–19)	0.780
Earning an income during past week				
Yes	207 (45.4)	101 (43.5)	106 (47.3)	0.417
No	249 (54.6)	131 (56.5)	118 (52.7)	
During past week, hours spend on paid work				
1–5	170 (79.4)	86 (81.1)	84 (77.8)	0.411
6–10	33 (15.4)	14 (13.2)	19 (17.6)	
11–15	5 (2.3)	3 (2.8)	2 (1.9)	
16–20	2 (0.9)	2 (1.9)	0 (0.0)	
21+	4 (1.9)	1 (0.9)	3 (2.8)	
Ownership personal mobile phone				
Yes	240 (52.1)	138 (58.5)	102 (45.3)	0.005
No	221 (47.9)	98 (41.5)	123 (54.7)	
(If no)Use of someone else’s mobile phone				
Yes	154 (69.1)	62 (62.6)	92 (74.2)	0.063
No	69 (30.9)	37 (37.4)	32 (25.8)	
(if yes) Sharing of personal mobile phone				
Yes	45 (18.8)	25 (18.2)	20 (19.6)	0.79
No	194 (81.2)	112 (81.8)	82 (80.40	
Internet access on mobile phone				
Yes	332 (84.5)	166 (83.0)	166 (86.0)	0.41
No	61 (15.5)	34 (17.0)	27 (14.0)	
Internet access in the past 6 months				
Yes	311 (67.5)	164 (69.8)	147 (65.0)	0.277
No	150 (32.5)	71 (30.2)	79 (35.0)	
Source of internet access				
Mobile Phone	283 (90.4)	146 (88.0)	137 (93.2)	0.449
PC	9 (2.9)	5 (3.0)	4 (2.7)	
Laptop	13 (4.2)	10 (6.0)	3 (2.0)	
Tablet	6 (1.9(	4 (2.4)	2 (1.4)	
Other	2 (0.6)	1 (0.6)	1 (0.7)	
Download App on phone				
Yes	335 (86.1)	171 (86.4)	164 (85.9)	0.887
No	54 (13.9)	27 (13.6)	27 (14.1)	

### Lessons learned

#### 1) Studies need to provide most participants with smartphones. These cannot be entry-level phones given the technological requirements of the survey application

**Fig 3:**
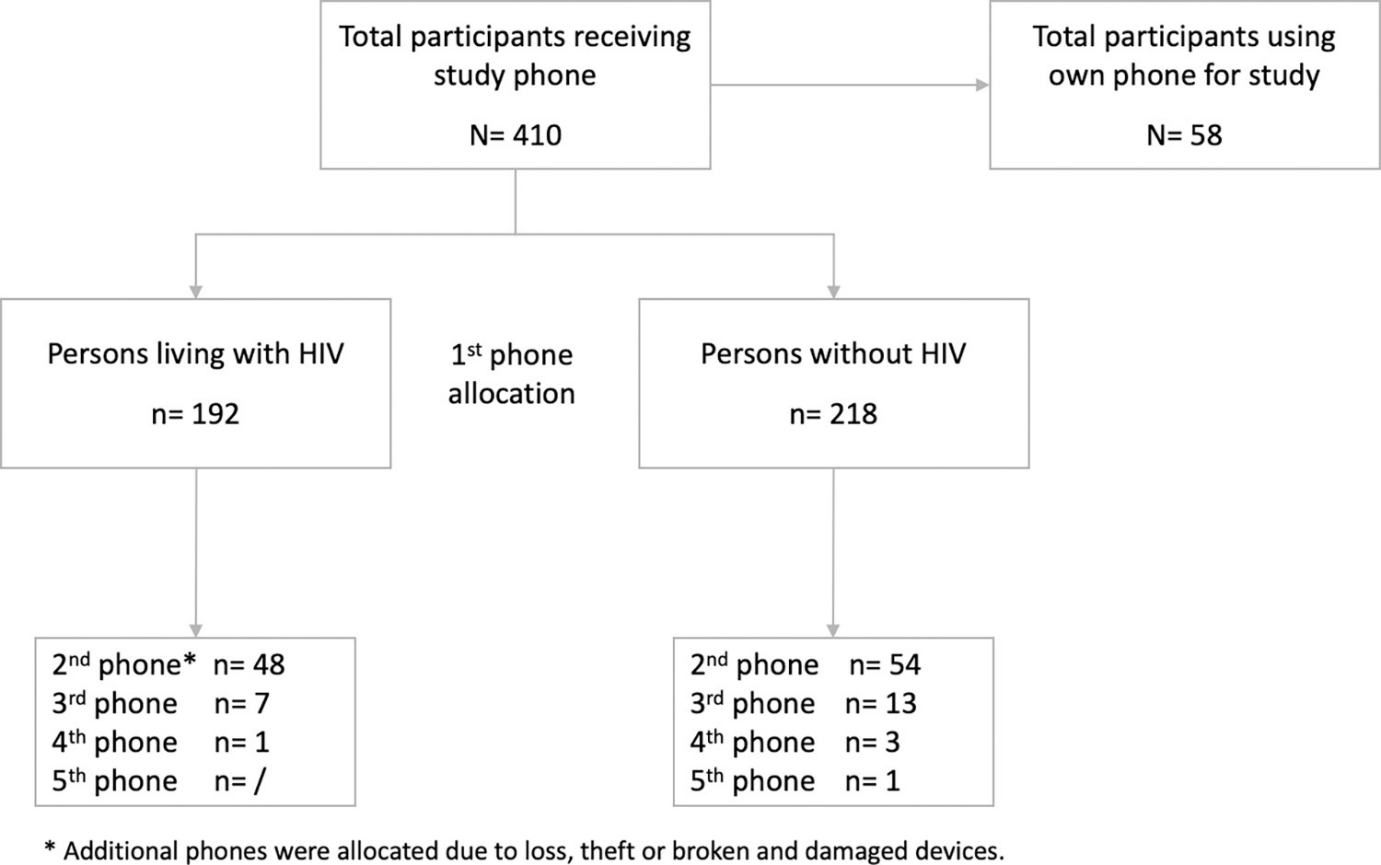
Study phone allocation flow chart.

Study phones were provided to participants who did not have a smartphone or did not own a phone that met the minimum software and hardware requirements to run the SurveyCTO app. The study team quickly realised that although participants owned or had access to a mobile phone, many young men did not own an adequate smartphone. Study phone allocation, for the period December 2020 to April 2022, was 87% (see Fig 3). Of 468 participants enrolled at this time, 410 required study phones. In addition, participants were provided with a replacement study phone when the personal or initial study phone had been lost, damaged, stolen, the app never functioned or the phone stopped working. The study originally purchased entry level Android smartphones that met the basic requirements for operating the SurveyCTO app with the survey form. However, the phones required many system updates over time, which slowed down the set-up of the phone, the SurveyCTO app, and survey submission. Therefore, the study team purchased better quality phones with updated Android operating systems and tested different smartphone brands to ensure adequate operation. Finally, the mobile phone network connectivity of the participants was reviewed during enrollment. If it was not adequate, a SIM card was purchased through a mobile phone provider network with better connectivity.

#### 2) Participants require ongoing, multimodal technological support

Participants encountered a myriad of technical challenges preventing them from submitting surveys. Participants communicated these through direct requests with the mobile phone educators, in follow-up calls with first time and non-submitters, and during the qualitative interviews. The main support requests included: forgetting their app pin and/or individual survey passcode, challenges with using the SurveyCTO app, lack of sufficient data (provided by the study) and poor network connection to submit the weekly survey through the SurveyCTO app, phone issues (i.e. phone being slow, freezing, or broken), and reporting a lost phone. Some participants stated:

“*The person that explained*, *explained it very well but when I opened my phone…I struggled with the pin thing*…*So I entered it at the end and then I placed the pin*. *There was a problem because I did not [find] my paper where they wrote the pin for me*.*”* ***(IDI-02-16 years)***

“*The app [CTO app] was troublesome*, *when I would try to send the [survey] form*. *It would just stay there and not be sent*.*”* **(IDI-03-16 years)**

***Interviewer:** “Why do you think some participants don’t always respond to the surveys, like they don’t answer the surveys?”*

***Participant:** “Because lack of… lack of … when there is no network.” **(IDI-016-18 years)***

In response to participant challenges, the SurveyCTO app and survey completion training was intensified, and more time was taken during the enrollment visit to ensure that participants understood each step of the survey submission process. WhatsApp was also downloaded onto the phone at enrollment for receiving quick study support. WhatsApp was chosen due to the popularity and frequency of use among South African youth. As a last step during the training component, the mobile phone educators saved important study numbers, including the study support WhatsApp number.

From the beginning of the study, telephonic check-in and support was provided to first time submitters after the enrolment visit as a quality assurance measure, to identify and resolve any challenges early, and to provide additional SurveyCTO app survey training where required. In addition, when real-time data monitoring revealed low survey submission rates, we began calling to check in with all participants who had not submitted two or more surveys consecutively. During these calls, technical support was provided, and training points were identified and resolved to encourage survey submission.

The original survey included a counsellor support request button at the end. However, many participants mis-used this to access technical support. Thus, a separate technical support request button was added at the end of survey form. This initiated request sent an SMS to the study technical support phone, and prompted a call from the staff. After implementing the technical support button, staff noted a high volume of technical requests that were placed by mistake. Thus, a confirmation button for technical support was then added, which streamlined the requests and reduced errors.

#### 3) Adolescent participants respond better to peer communication via WhatsApp or text, as compared to phone calls from staff

In the process of improving our various technical support strategies as well as improving the response rates of the surveys, a male peer-educator to advise on study processes and oversee participant engagements activities (e.g., WhatsApp broadcast messages and content) was recruited. This peer-educator was well placed to respond to additional requests voiced by participants during the in-depth interviews: these included receiving regular study check-ins, study updates, reminders on the weekly survey submission process, and reminders of the support available. We found that the best way for the peer-educator to communicate was by using the anonymous WhatsApp broadcast messaging service. In addition, community engagement was intensified by including a young male CAB member on the bi-weekly operational Tsamaisano team calls to get insights and feedback on study related updates.

#### 4) Facilitating a guided discussion around mobile phone access and survey completion during the enrolment visit can help ensure participants fully understand the commitment required

There was a substantial number of participants with very low to no survey completion, despite the above efforts. Thus, a greater attempt was made to ensure enrollment of ‘best fit’ participants: adolescents had to understand and express commitment to the mobile component of the study as well as report sufficient mobile phone usage (an indicator they would likely submit surveys). To ensure that both the adolescent participant and their caregiver understood the commitment required for successful EMA, we created and implemented a guided discussion around mobile phone access during the enrolment visit. The team recommended that it was critical to involve the caregiver, as they often control access their child’s mobile phone, particularly when disciplining their child. The outcome of the discussion was an agreement between the caregiver and the child that facilitated survey submissions, such as when participants should check their phones for survey requests, and how punishments should be managed to allow for submissions. Moreover, some adolescents shared a phone with other family members, which could make it difficult to track survey request and reminder SMSs and submit on time. Through this more nuanced discussion, staff could assess whether a dedicated study phone should be provided. Survey completion commitment was reiterated throughout the enrolment visit and through a written commitment. These additions to the eligibility criteria for study participation were reviewed and approved by the institutional review boards at University of the Witwatersrand and Stony Brook University

#### 5) Participants’ non-responses and preferring a fixed survey submission day to a random submission day

During the interview feedback sessions, participants reported reasons for not submitting surveys: it was boring to answer the same weekly questions; they found it difficult to answer sensitive questions about violence and sex; and they simply forgot to submit surveys. Some participants stated: *“I can say some find it boring … so that is why they answer late or maybe just leave it like that [not answer the survey at all]*.*”* ***(IDI-02-16 years)***

“*Maybe another reason can be the same questions every week*.*”* ***(IDI-011-17 years)***

In response to questions about random versus set submission days, participants stated that it would be easier to remember a fixed weekly submission day. They stated a fixed day would allow them to build a routine over time. For example some reported:

“*It’s Friday [Best day to receive the survey] …Yes*, *I knock off early at school so I have plenty of time…5pm…Because that is when I am no longer doing anything*, *I am just busy with my phone or watching TV or I could just go out*. ***(IDI-07-19 years)***

“*It is better Thursday*, *so it is better that Thursday I didn’t answer the survey [In case he forgot to answer the survey on Thursday]*. *Friday I know that I am home*, *they call me*, *they find me they remind me then I do it*. ***(IDI-03-16 years)***

**Fig 4:**
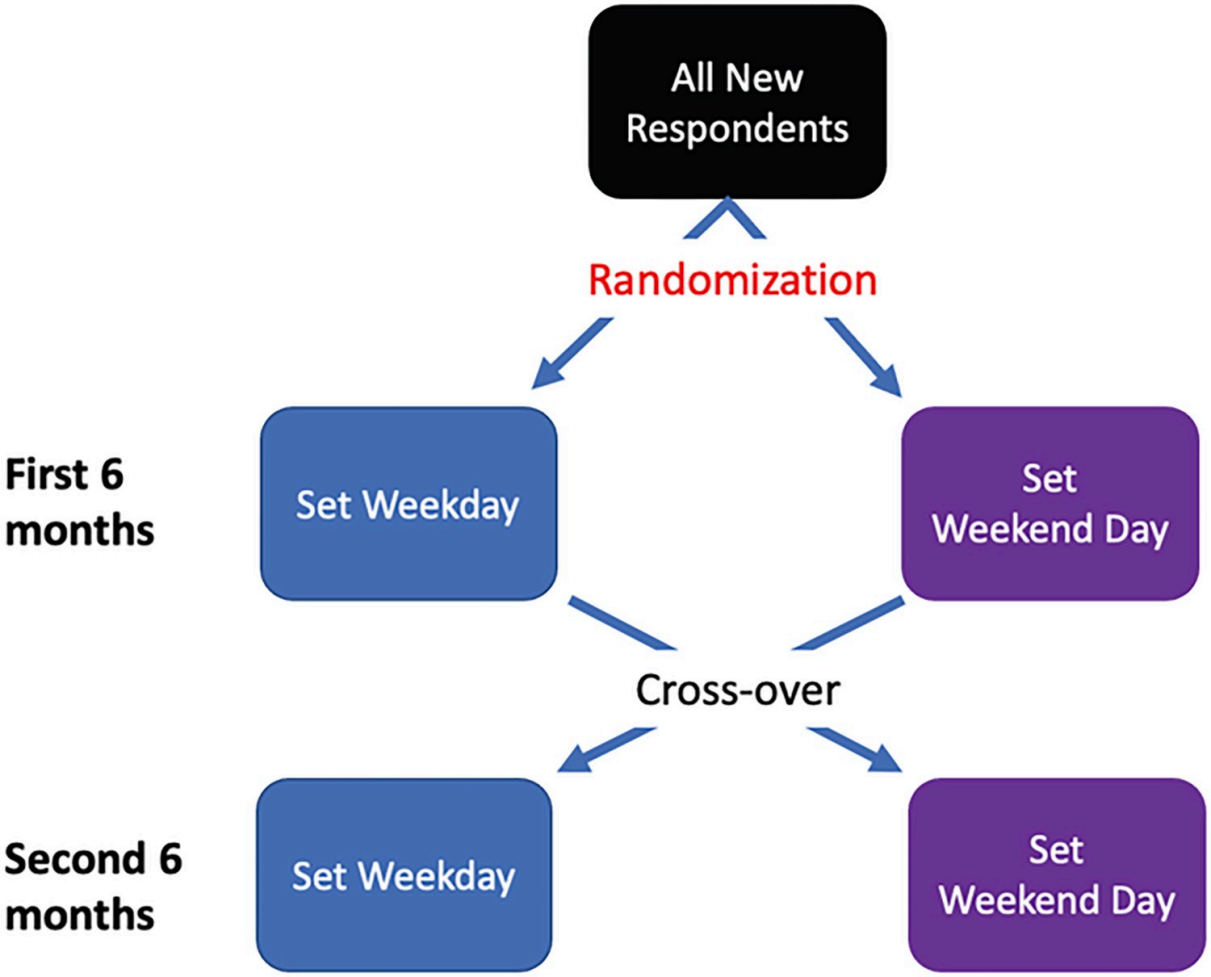
Revised schedule for allocating mobile survey submission requests.

To ensure that the study still had adequate coverage from all days of the week, we took the following steps starting in July 2021: one half was assigned to a randomly selected set week day and the other to a randomly selected set weekend day. After six months, those who were randomly assigned to a weekday for the first six months were then assigned a weekend day for the second six months and vice versa (see Fig 4). This assured adequate coverage of all seven days for the study, while still enabling the participants to establish an individual routine around a set survey day.

Participants also suggested reminder messages be changed, and suggested times that they would be most likely see them. The excerpts below illustrate participant suggestions:

“*Maybe I would want it [reminder SMSs] to say*, *‘You have less than an hour to submit the survey*.*’ …Or ‘One day left to submit*.*’ Or ‘Don’t forget to submit the survey today*.*”* ***(IDI-09-19 years)***

“*It will be easy if they maybe change it [SMS] and say*: *‘It’s time to answer’…”****(IDI-010-16 years)***

The SMS times were changed from receiving the survey request SMS in the afternoon to receiving the request SMS in the early morning (i.e. 6:00), followed by reminders in both the same day afternoon (17:00) and the following day afternoon (17:00). The wording for the SMSs were revised on participant request to be more instructive on when surveys had to be submitted to still be eligible for the R10 airtime incentive. Additionally, the survey request SMS also informed participants that their data bundle had been loaded. In feedback sessions, participants noted that receiving data prior to the survey completion and a small airtime incentive after successfully submitting a survey record were motivators to continue submitting surveys. When asked about alternative incentives, most said they were satisfied with the current arrangement.

#### 6) A clear strategy for delivering data is needed to enable survey submissions, particularly among low-income youth

Submission of surveys requires data. Among South African young people from low-income settings, unlimited data plans are rare. In our context, the majority owned mobile phones requiring pre-paid airtime and data and the study phones require the same. At the beginning of the study, 100 MB of data was automatically sent to participants together with the first survey submission request. As noted earlier, the SMS survey request timing was changed to the early mornings to correspond to the participants wake up time. This was to ensure that participants had a full first day to submit their surveys. However, in both instances, participants often used their allocated data volume prior to submitting their survey, which resulted in non-submission or completed surveys being stuck in the outbox of the SurveyCTO app as they could not be successfully transmitted without mobile phone data. In response, the 100 MB (~$1.5) data allocation was split to send in the morning and the remainder data allocation in the afternoon. This increased the likelihood that participants would have enough mobile data to successfully submit their survey if they chose to do it in the afternoon.

#### 7) Studies need to be prepared for the large volume of psychosocial support

Importantly, participants mentioned that they appreciated that the survey was anonymous and therefore they could answer sensitive questions, including those around violence and sex, without judgments by a study staff member. However, some participants reported that it was difficult answering sensitive questions every week, especially when it was connected to one’s own recent experiences or that of a close family or community member. This underscores the need for robust psychosocial support. Below are quotes stating participants reports on answering the surveys.

“*This survey is thought provoking because there are questions which made me realise and think that things are happening to people… You see but I would have a question on how they solve this problem and I realise that I think this is the best place to solve issues like physical abuse*.*”* ***(IDI-07-19 years)***

“*The questions for me are clear but it is up to me how I respond to them know*. *It becomes difficult to answer them*. *You find that the question is asking something that you have experienced and you find that the question takes you back to that space … Because I think about all the things that I used to witness when I was still young*.*”* ***(IDI-07-19 years)***

For all participants enrolled in the surveys, support was available 24 hours a day. At the end of the survey, there was a button that the participant could use to request psychosocial support. Once initiated, an automated SMS was sent with the local contact telephone numbers for service organizations, including Childline [39] and the South African Depression and Anxiety Group [40]. Additionally, an automated SMS was sent to the social worker or psychologist on call. They would contact the participant to debrief and/or arrange services. Finally, a question within the survey on being physically hurt or being sexually assaulted was programmed to trigger an automated notification to the study team, promoting a counsellor or social worker to reach out. If a participant revealed that they were in immediate danger or a danger to others, they were brought into the clinic for urgent support, referred to any necessary additional services, and the South African PI was notified. While we anticipated the need for these services, we did not anticipate the volume of need. Many participants utilized the counsellor request button within the survey. Since the separate technical support button was implemented from the week of 22 February 2021, on average, of the 9502 submitted surveys, 256 (2.7%) requested a counsellor, 712 (7.5%) were flagged to have reported being physically hurt and/or sexually assaulted and 1179 (12.4%) requested technical support.

Table 2 provides an overview of barriers to survey completion and recommendations with illustrative key quotes from IDI participants.

**Table 2:** Barriers to survey completion and recommendations.

Theme: Barriers to survey completion	
Sub-themes	Key Quotations from Qualitative Data
Forgetting to answer the survey and finding it boring to answer the same questions weekly.	*“I can say some find it boring … so that is why they answer late or maybe just leave it like that [not answer the survey at all]*.*”* ***(IDI-02-16 years)*** *” … Maybe another reason can be the same questions every week*.*”* ***(IDI-011-17 years)*** *“I forgot that I have Tsamaisano*. *That was at the beginning … I forgot that … there is a survey that I need to complete … so the data*, *I used it*. *I didn’t do anything …*.*”* ***(IDI-08-16 years)***
Technical issues prevented survey completion.	*“The person that explained*, *explained it very well but when I opened my phone…I struggled with the pin thing*.* *.* *.*So I entered it at the end and then I placed the pin*. *There was a problem because I did not [find] my paper where they wrote the pin for me*.*”* ***(IDI-02-16 years)*** *“The app was troublesome*, *when I would try to send the [survey] form*. *It would just stay there and not be sent*.*”* **(IDI-03-16 years)** *“I forgot the password…which is*, *it was three letters but when [then] I lost my phone…So I had to make a new one [download the app again] so I forgot the password*.*”* ***(IDI-07-19 years)*** ***I*:** *“Why do you think some participants don’t always respond to the surveys*, *like they don’t answer the surveys*?*”* ***R*:** *“Because lack of… lack of … when there is no network*.*”* ***(IDI-016-18 years)***
Difficulty in answering sensitive questions about violence and sex.	*“This survey is thought provoking because there are questions which made me realise and think that things are happening to people… You see but I would have a question on how they solve this problem and I realise that I think this is the best place to solve issues like physical abuse*.*”* ***(IDI-07-19 years)*** *“The questions for me are clear but it is up to me how I respond to them know*. *It becomes difficult to answer them*. *You find that the question is asking something that you have experienced and you find that the question takes you back to that space … Because I think about all the things that I used to witness when I was still young*.*”* ***(IDI-07-19 years)***
**Theme: Recommendations to improve survey completion**	
Set survey completion days (as opposed to random allocation) to build a routine over time	*“Saturday and Sunday [best time to answer the survey] …Because as I am in matric*, *I attend every day at school and so sometimes it happens that I carry my phone to school because I want to do research…So I would get phone calls while still in class…And there’s a teacher*, *so…It becomes a problem*.*”* ***(IDI-05-19 years)*** *“It’s Friday [Best day to receive the survey] …Yes*, *I knock off early at school so I have plenty of time…5pm…Because that is when I am no longer doing anything*, *I am just busy with my phone or watching TV or I could just go out*. ***(IDI-07-19 years)*** *“It is better Thursday*, *so it is better that Thursday I didn’t answer the survey [In case he forgot to answer the survey on Thursday]*. *Friday I know that I am home*, *they call me*, *they find me they remind me then I do it*. ***(IDI-03-16 years)***
Request and reminder SMSs for survey completion	*“Maybe I would want it [reminder SMSs] to say*, *‘You have less than an hour to submit the survey*.*’ …Or ‘One day left to submit*.*’ Or ‘Don’t forget to submit the survey today*.*”* ***(IDI-09-19 years)*** *“It will be easy if they maybe change it [SMS] and say “It’s time to answer…”****(IDI-010-16 years)***

## Discussion

We describe key lessons learned to inform future study designs with mobile phone EMAs, drawing on our experience implementing such among adolescent boys in a low-and-middle income setting. Our lessons point to the WHO youth-centered digital health interventions framework, to ensure that users are included throughout the research life cycle by leveraging youth-adult partnerships [41]. More than half of the participants required study phones, which was more than was expected, but perhaps is a more realistic picture among participants from lower socio-economic settings. The high volume of technical support requests included common problems that could arise in any setting (e.g., forgetting pins and passwords, pressing the technical request button in error) as well as unique barriers specific to those from low-socio-economic settings (e.g. owning lower end android phones, poor network connectivity, low data access).

Mobile and remote data collection have proven to be feasible but are also not without challenges. A review of low and middle-income countries revealed that the major challenges included: network shortcomings, the need to constantly train users, and the collection of sensitive information [15,42]. Furthermore, a recent Soweto study with similar data collection methods revealed other important limitations of using mobile phones; there were challenges around phones getting lost or breaking during the duration of the study, which resulted in the issuing of new phones [14,15]. Similar challenges were observed during our study, which had to be addressed and strategies developed in an ongoing process. However, many studies evaluating mobile-technology based EMA methods, including with young people, do not specifically report on technical and logistical challenges [18]. This study adds value to the existing literature around using mobile EMA amongst young people from low socio-economic backgrounds, as we describe both technical and logistical challenges since study inception as well as adaptations performed.

Young men in our study reported response fatigue to the same weekly questions as one of the reasons for non-response of the surveys. Response fatigue has been observed when collecting survey data and can stem from various reasons, such as completion length, questions topics, survey questions complexity and type [43]. Short surveys with only a few questions can reduce respondent fatigue [14]. Even though the survey in our study is short and simple, some participants still reported boredom with answering the same weekly survey. For future studies incorporating EMA could explore youth-friendly design to ensure study engagement over time.

In our study, participants expressed a preference for set weekly survey days versus randomly selected days. They stated it would help them better fit the survey completion into their schedule and be able to add their own individual reminders over and above the study SMS reminders. In another study conducted in Soweto, young women who completed a daily survey over a period of 90 days, with an overall response rate of 82%, also reported implementing their own reminder strategies. This included setting an additional alarm on their phones or building the daily survey completion into their morning routine [14].

Communication with participants was important for multiple aspects of study implementation, including survey completion, technical support, retention and engagement. Initially, phone calls and text messages were used as the primary medium of communication. However, WhatsApp was a more successful strategy to increase communication between the study site and participants. In South Africa, WhatsApp is the most popular messaging application used for socialization, business, educational purposes and health interventions. In a population of 58.93 million people, 38 million South Africans are active WhatsApp users [44]. The General Data Protection Regulation stipulates the minimum age for WhatsApp use in South Africa is 13 years [45]. The 2015 student tech survey showed that 92% of South African students were WhatsApp users [46]. Through Tsamaisano, we started a WhatsApp broadcast group, to transmit important news, updates, and check-ins with the aim to increase study engagement and improve survey submission performance. The WhatsApp broadcast function ensured privacy because those receiving the broadcast messages cannot see the contact details of those receiving the messages.

### Limitations

Although the data for the interviews can not necessarily be generalized to all the Tsamaisano participants, the data are in agreement with previous research conducted in Soweto and data saturation was reached [13,14]. Further, our data are restricted to adolescent boys, lessons can be applied to youth in low-income South African settings. Although many the findings are applicable to mobile phone EMA studies in general, some of the technical challenges related to are specific to the SurveyCTO app. Our study did not apply a specific youth-centred framework but included youth engagement strategies. Future EMA studies involving reporting of sensitive information could incorporate a youth-centred framework upfront to guide and inform the implementation of the EMA design [47]. Our lessons point to the WHO youth-centered digital health interventions framework, to ensure that users are included throughout the research life cycle by leveraging youth-adult partnerships.

## Conclusions

We describe key lessons learned to inform future study designs with mobile phone EMAs, drawing on our experience implementing such among adolescent boys, including persons living with HIV, in a low-and-middle income setting. The key lessons learned through the Tsamaisano study are important to inform future study designs with EMA using mobile phones, electronic data collection among adolescent boys in low-and-middle-income settings. Participants expressed a preference for set weekly survey days versus randomly selected days. We identified a need for increased and ongoing technical support; addressed by creating technical guides, implementing a standard two-week check-in call after enrolment, adding an automated request button for call-back assistance, creating a WhatsApp messaging stream, and proactively reaching out to all participants failing to submit two sequential surveys.
